# Idiopathic Mast Cell Activation Syndrome With Associated Salicylate Intolerance

**DOI:** 10.3389/fped.2018.00073

**Published:** 2018-03-27

**Authors:** Tobias Rechenauer, Martin Raithel, Thomas Götze, Gregor Siebenlist, Aline Rückel, Hanns-Wolf Baenkler, Arndt Hartmann, Florian Haller, André Hoerning

**Affiliations:** ^1^Department of Pediatrics and Adolescent Medicine, University Hospital Erlangen, Friedrich Alexander University Erlangen-Nuremberg, Erlangen, Germany; ^2^Medical Clinic II, Waldkrankenhaus, Erlangen, Germany; ^3^Medical Clinic III for Rheumatology and Immunology, University Hospital Erlangen, Friedrich Alexander University Erlangen-Nuremberg, Erlangen, Germany; ^4^Institute of Pathology, Friedrich Alexander University Erlangen-Nuremberg, Erlangen, Germany

**Keywords:** idiopathic mast cell activation syndrome, mast cell activation, salicylate intolerance, mastocytosis, endoscopically guided lavage, abdominal pain

## Abstract

Idiopathic mast cell activation syndrome can be a rare cause for chronic abdominal pain in children. It remains a diagnosis by exclusion that can be particularly challenging due to the vast variety of possible clinical manifestations. We present a 13-year-old boy who suffered from a multitude of unspecific complaints over a long period of time. In this case, an assessment of mast cell-derived metabolites and immunohistochemical analysis of bioptic specimen was worthwhile. After ruling out, primary (oncologic) and secondary causes for mast cell activation, pharmacologic treatment adapted to the patient’s salicylate intolerance resulted in a major relief of symptoms.

## Background and Case Presentation

Chronic abdominal pain in children and adolescents is a common cause for medical consultations and can be associated with other non-specific symptoms such as headaches and nausea. Even if no organic cause is obvious in routine examination and laboratory checkup, sometimes a more profound investigation can be worthwhile.

A 13-year-old male adolescent presented with a history of suffering from paroxysmal spasmodic bowel pain, nausea, dizziness, headaches, and hot flashes for a period of over 2 years. Due to his complaints he missed over 12 weeks of school and also reported a loss of sleep. The family was not aware that the symptoms were associated with any certain circumstances or a particular diet. Preceding explorations for basic laboratory blood analysis, celiac serological testing, stool examination for bacteria, parasites, and chronic inflammatory bowel disease (repeatedly erythrocyte sedimentation rate, C reactive protein, and fecal calprotectin), allergy checkup (serological IgE-RAST for foods and skin Prick testing), abdominal and cardiac ultrasound, electroencephalography, and cranial MRI imaging were without pathological findings. Two previous gastric endoscopies only found a minor unspecific gastritis without evidence for a *Helicobacter pylori* infection, and it was treated with proton pump inhibitors without any reported improvement of symptoms. Intestinal carbohydrate malabsorption (fructose, lactose, and sorbitol breath tests) was excluded. A symptom diary finally showed an association to the consumption of cheese and chocolate, possibly hinting at histamine intolerance. Furthermore, the patient reported a slight relief of symptoms while implementing a histamine-reduced diet, thus a double-blind placebo controlled food challenge for histamine was planned. Salicylate intolerance was suspected due to reported complaints and diagnosed by implementing an extended functional eicosanoid test determining the release of the eicosanoids leukotrienes and prostaglandines after *in vitro* exposure to salicylates.

**Figure 1:**
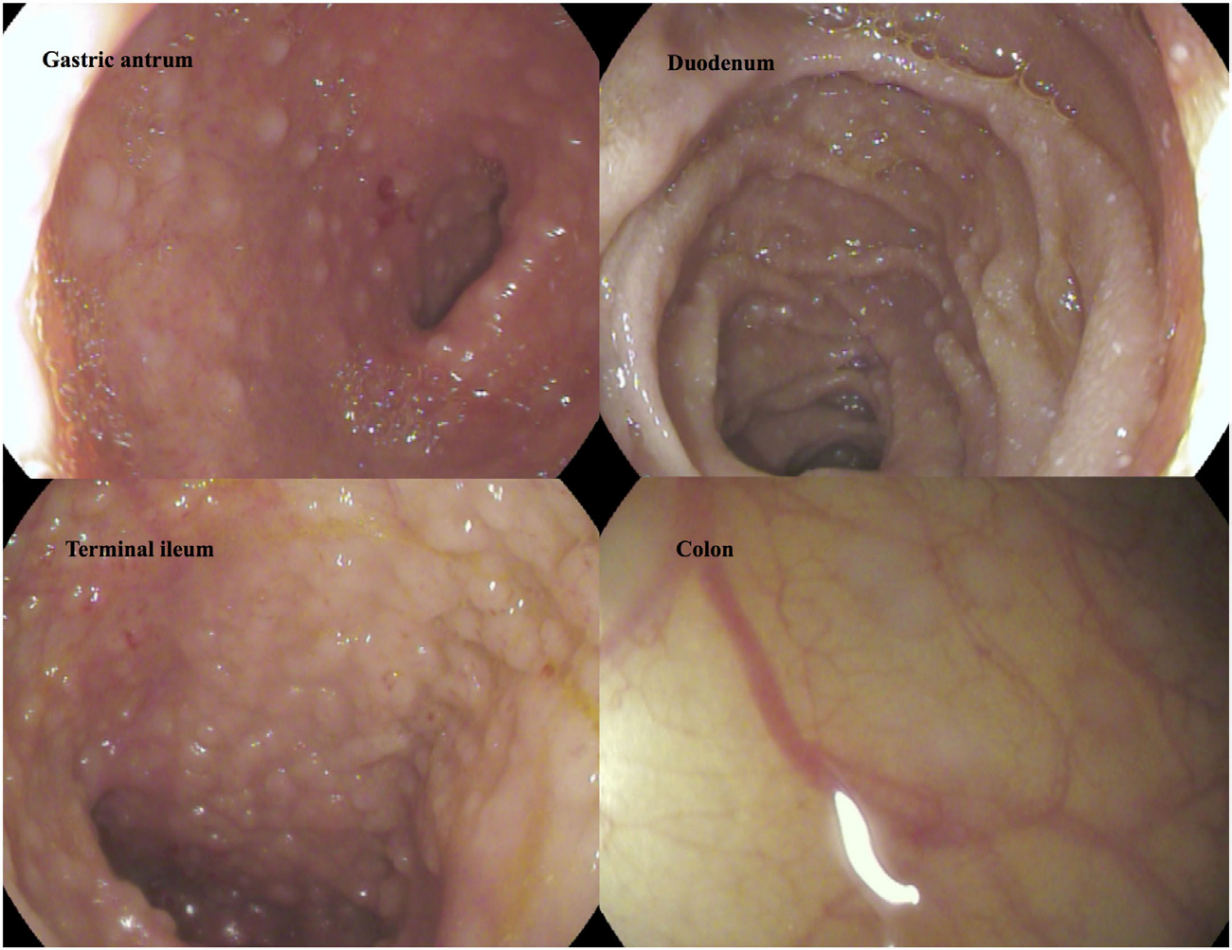
Macroscopic findings in gastroscopy and colonoscopy. Follicular hyperplasia in upper and lower intestinal tract.

**Figure 2:**
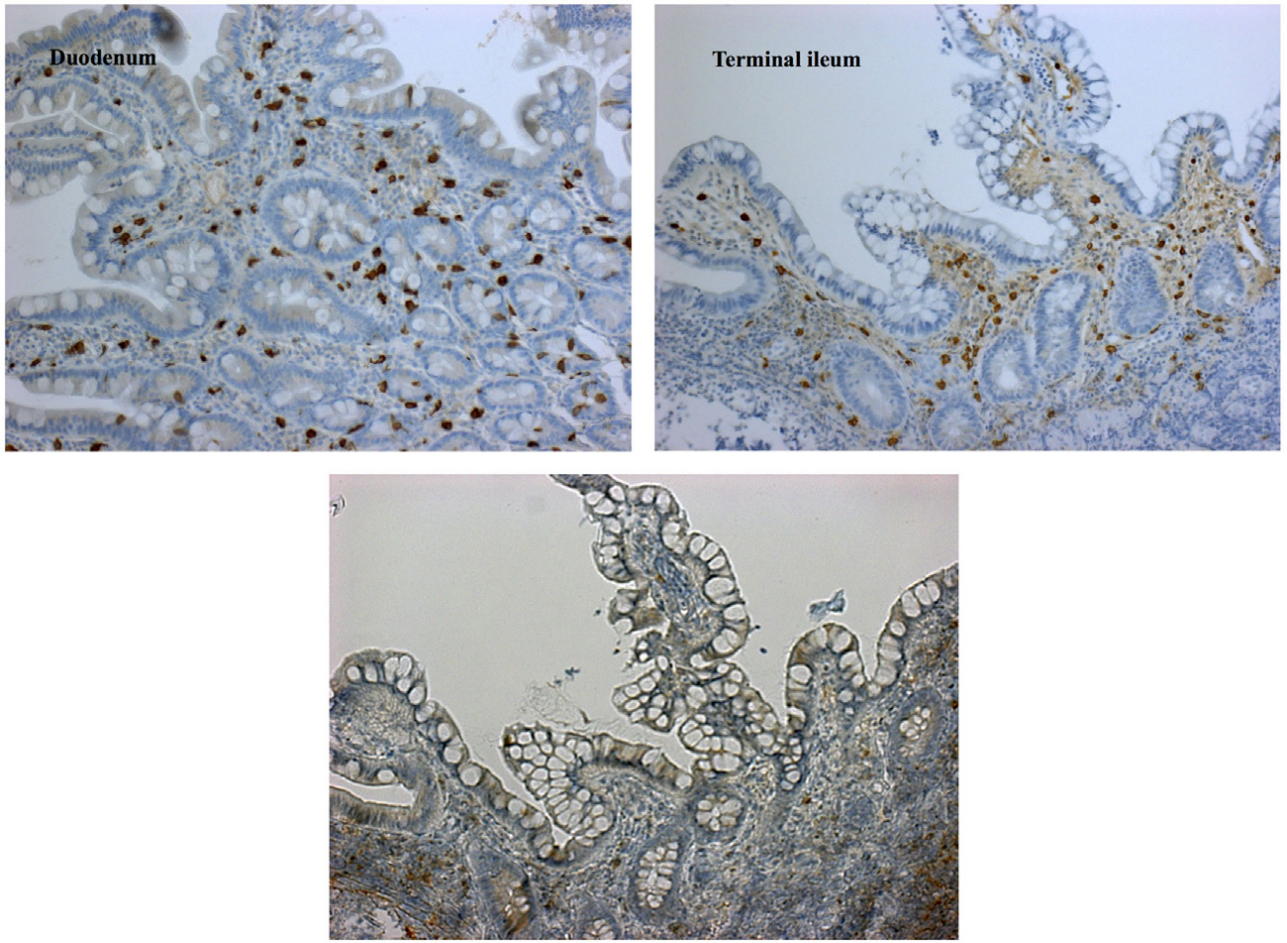
Immunohistochemical findings demonstrating mature mast cell infiltration. Dense stromal mast cell infiltrations of up to 60/HPF [staining for CD117^+^ (left), tryptase (right), and CD25^−^ (lower left image)]; mature type; cKIT-D816V mutation negative; normal crypt architecture; no eosinophils.

Although levels for tryptase (2.7 µg/L) were in normal range, repeatedly elevated serum tumor necrosis factor alpha (TNFα) ranging from 10.5 to 27.7 pg/mL (ref. <8.1 pg/mL) as well as significantly enhanced excretion of methylhistamine (17.8 µg/mmol crea/m^2^ BSA; ref. value <6.5 µg/mmol crea/m^2^ BSA) in a 24 h urine sample indicating a state of high endogenous histamine production (1). The endoscopic evaluation macroscopically showed nodular hyperplastic spots particularly in the gastric antrum and terminal ileum (Figure 1). Hematoxylin–eosin stains from upper and lower GI tract revealed duodenal, ileal, and colonic lymphoplasmacytic infiltration, but no signs of erosive inflammation or epithelial damage. Again, an infection with *H. pylori* was histologically ruled out in the biopsy specimen of the gastric antrum. Immunhistochemically, up to 60 tissue mast cells per high power field with normal morphology were detected (no immature cell forms or spindle shapes, CD117^+^, MCT^+^, CD25^−^, and c-KIT D816V negative; Figure 2). In a segmental endoscopically guided lavage from the lower GI tract, a significant intestinal (local) IgE secretion specific to common food antigens was not detected, but histamine degradation capability was found to be modestly reduced in all specimen (Table S1 in Supplementary Material) (2).

Since all diagnostic criteria for idiopathic mast cell activation syndrome (IMCAS) as suggested by Akin et al. (3) and Valent et al. (4) were met (Table 1), the patient received nutritional counseling to avoid histamine and salicylate rich foods and oral treatment with histaminic receptor blockers (ketotifen 1× 1 mg), a leukotriene antagonist (montelukast 1× 4 mg), and slow-release vitamin C (1× 500 mg) to inhibit mast cell degranulation (5). A possible treatment option with cromoglicic acid was avoided due to intolerance to salicylates. Melatonin 2 mg was given orally to treat sleep disturbances.

**Table 1:** Diagnostic criteria for idiopathic mast cell activation syndrome.^a^

All four criteria must be fulfilled
Episodic symptoms consistent with mast cell mediator release affecting ≥2 organ systems (skin, gastrointestinal, cardiovascular, respiratory, naso-ocular, and neuropsychiatric^b^) Response of clinical symptoms to histamine receptor blockers or mast cell targeting agents Evidence of an increase in validated urinary or serum markers of mast cell activation (MCA) Rule out of primary (oncologic) and secondary causes of MCA

*^a^Slightly abbreviated and modified from the original versions by Akin et al. (3) and Valent et al. (4)*.

*^b^Typical symptoms, e.g., flushing, pruritus, urticaria, angioedema, nasal congestion and conjunctival injections, arterial hypotension and tachycardia, syncope, diarrhea, abdominal cramping, headaches, fatigue, and pulmonary wheezing*.

Under this regime, the patient experienced a major improvement of complaints, no side effects were observed. Administering medication before going to sleep averted a possible ketotifen-associated fatigue.

## Discussion

Idiopathic mast cell activation syndrome is a relatively “new” disorder as it was first described in 2010 (3, 4). It is used to describe symptoms caused by mast cell activation (MCA) that cannot be explained by an underlying allergic or inflammatory condition and do not fulfill the WHO criteria for oncologic conditions such as systemic or cutaneous mastocytosis (6, 7). The latter are rare diseases that involve clonal expansion of mutated stem cells and are not to be confused with IMCAS. Therefore, a thorough clinical examination for urticarial pigmentosa as an indicator for cutaneous mastocytosis and laboratory assessment for elevated tryptase levels (particularly >20 ng/mL), CD2, CD25 expression and c-KIT_D816V mutation in mast cell infiltrates is essential (6, 7). In bioptic tissue specimen of IMCAS multifocal, dense infiltrations with atypical morphology, immature, or spindle shaped mast cells should not be present. In case of uncertainty weather an underlying clonal disease is present bone marrow biopsy and molecular genetic analysis for c-Kit mutations should be considered.

Idiopathic mast cell activation syndrome remains a diagnosis by exclusion that can be very challenging to assess due to multitude of possible clinical manifestations of symptoms and etiologies leading to MCA. Clinical symptomatology is derived from an increase of mast cell mediators and can be classified to organ systems (Table 1). In a state of increased MCA, an elevation of validated markers such as serum tryptase, TNFα, 24-h urine histamine metabolites, PGD, or 11-β-prostaglandin F_2_ are essential for diagnostic approach (3, 4). The difficulty to diagnose IMCAS is to demonstrate signs of definitive MCA while excluding secondary etiologies explaining mast cell involvement such as allergy, infection, autoimmunity, and tumors.

In our case, a segmental endoscopically guided lavage from the lower GI tract was made to detect local IgE production. This enabled us to exclude local seronegative food allergy as a relevant cause of mast cell accumulation and activation.

The treatment options for IMCAS include the avoidance of identifiable triggers for mast cell degranulation, histamine receptor antagonists, mast cell stabilizing substances such as cromoglicic acid, leukotriene receptor blockers, possibly glucocorticoids, and slow-release vitamin C (inhibition of mast cell degradation and activation of histamine degradation) (8). Response to pharmacologic treatment also is a diagnostic criterion for IMCAS (Table 1).

Salicylate intolerance refers to an altered metabolism of arachidonic acid and eicosanoids leading to a predominance of leukotrienes over prostaglandins. The main subgroups of eicosanoids—the leukotrienes and the prostanoids with the prostaglandins and thromboxanes—are predominantly formed by the lipoxygenases and the cyclooxygenases, respectively. In intolerant individuals, activation of mast cells, basophiles and eosinophils, macrophages, platelets, and lymphocytes is common and well described (9). The pattern of symptoms may depend on location, relative numbers of the responsible cells, and mainly on the activation state of mast cells (10). As activated mast cells primarily express formation of eicosanoids after contact with salicylates besides histamine release and other mediators, salicylate intolerance may give a phenotypic advice for a possible underlying IMCAS.

## Concluding Remarks

In summary, especially for children with a long history of MCA-compatible symptoms, IMCAS must be considered after ruling out chronic inflammatory bowel disease or other primary and secondary causes. As IMCAS seems to be underdiagnosed for adults probably due to overlaps with irritable bowel diseases and fibromyalgia, the diagnosis for children is currently even scarcer. In fact, this might be one of the first case reports for salicylate intolerance and IMCAS in children.

## Ethics Statement

Written informed consent for the publication of the case report was obtained from both parents and the patient.

## Author Contributions

TR designed, drafted, and revised the case report. FH and AHartmann performed the histological analysis. MR assessed cytokine measurement in endoscopic lavage fluids and revised the case report. AHoerning performed the endoscopy, diagnosed and treated the patient, and revised the case report. TG, GS, and AR performed the endoscopy, intestinal lavage, and collection of bioptic specimen. H-WB analyzed the FET test and approved the result. All the authors approved the final manuscript and agree to be accountable for all aspects of it.

## Conflict of Interest Statement

The authors declare that the research was conducted in the absence of any commercial or financial relationships that could be construed as a potential conflict of interest.

## Abbreviations

MCA, mast cell activation; IMCAS, idiopathic mast cell activation syndrome; CD117, tyrosine kinase c-KIT; TNFα, tumor necrosis factor alpha; ESR, erythrocyte sedimentation rate; CrP, C reactive protein; PGD_2_, prostaglandin; FET, functional eicosanoid test; MCT, mast cell tryptase.
