# Supplementary material for: Idiopathic Mast Cell Activation Syndrome With Associated Salicylate Intolerance
Source: Front Pediatr. 2018 Mar 27;6:73. doi: 10.3389/fped.2018.00073 (PMC5881244; doi:10.3389/fped.2018.00073)
Supplement: Supplementary file 1 [file table_1.DOC]

**Supplemental table 1: Endoscopically guided lavage for (local) sero-negative food allergies**

|  | **Terminal Ileum (U/mg)** | **Coecum (U/mg)** | **Sigma (U/mg)** | **Ref. Range (U/mg)** |
| --- | --- | --- | --- | --- |
| **Total IgE** | 0.21 | 0.30 | 0.16 | < 0.35 |
| **Specific IgE** |  |  |  |  |
| Chicken egg | 0.04 | 0.02 | 0.03 | < 0.15 |
| Egg yolk | 0.10 | 0.04 | 0.06 | < 0.15 |
| Egg white | 0.02 | 0.02 | 0.04 | < 0.15 |
| Lupine | 0.02 | 0.01 | 0.00 | < 0.15 |
| Casein | 0.04 | 0.02 | 0.03 | < 0.15 |
| Lactalbumine | 0.04 | 0.01 | 0.03 | < 0.15 |
| nGal d2 Ovalbumin | 0.00 | 0.00 | 0.00 | < 0.15 |
| nGal d1 Ovomucoid | 0.00 | 0.00 | 0.00 | < 0.15 |
| Pig | 0.06 | 0.04 | 0.04 | < 0.15 |
| Beef | 0.17 | 0.12 | 0.12 | < 0.15 |
| Celery | 0.02 | 0.00 | 0.01 | < 0.15 |
| Rye | 0.10 | 0.05 | 0.09 | < 0.15 |
| Wheat | 0.15 | 0.07 | 0.12 | < 0.15 |
| Barley wheat | 0.08 | 0.05 | 0.04 | < 0.15 |
| Oat wheat | 0.06 | 0.04 | 0.06 | < 0.15 |
| Corn wheat | 0.15 | 0.07 | 0.07 | < 0.15 |
| Buckwheat | ***0.17*** | 0.09 | 0.07 | < 0.15 |
| Baker yeast | 0.08 | 0.05 | 0.06 | < 0.15 |
| Gluten | 0.02 | 0.01 | 0.01 | < 0.15 |
| Nut Mixture fx1 | ***0.25*** | 0.13 | ***0.16*** | < 0.15 |
| Nut Mixture fx22 | 0.00 | 0.00 | 0.00 | < 0.15 |
| rGly m4PR-10 | 0.02 | 0.00 | 0.00 | < 0.15 |
| Codfish | 0.02 | 0.01 | 0.01 | < 0.15 |
| Sesame | 0.13 | 0.08 | 0.09 | < 0.15 |
| Spice mixture fx71  cumin, nutmeg, cardamom, clove | < 0.10 | < 0.10 | < 0.10 | < 0.15 |
| Spice mixture fx72  fennel, basil, ginger, anise | < 0.10 | < 0.10 | < 0.10 | < 0.15 |
| Parsley | < 0.10 | < 0.10 | < 0.10 | < 0.15 |
| Sunflower seeds | < 0.10 | < 0.10 | < 0.10 | < 0.15 |
